# Comprehensive multi‐omics analysis provides biological insights and therapeutic strategies for small‐cell lung cancer

**DOI:** 10.1002/mco2.569

**Published:** 2024-05-29

**Authors:** Guo Zhao, Yuning Wang, Shuhang Wang, Ning Li

**Affiliations:** ^1^ Clinical Trial Center, National Cancer Center/National Clinical Research Center for Cancer/Cancer Hospital Chinese Academy of Medical Sciences and Peking Union Medical College Beijing China

## Abstract

Integration of multi‐omics analysis into small‐cell lung cancer (SCLC) research. In the research of small‐cell lung cancer, the integration of multi‐omics analysis has become an important research direction. Multi‐omics analysis includes the study of genomics, transcriptomics, proteomics, metabolomics, and other levels, which can help us to understand the pathogenesis and development process of diseases more comprehensively as well as develop novel therapeutics and biomarkers for further precision oncology.

## Graphical abstract

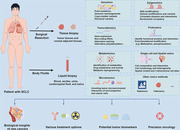

1

A recent study published in *Cell* by Liu et al.^1^ revealed comprehensive multi‐omics characterization (genomics, transcriptomics, proteomics, and phosphoproteomics) of small‐cell lung cancer (SCLC) using resected tumor tissues and corresponding normal adjacent tissues obtained from 112 untreated Chinese patients. The study first uncovered genetic abnormalities of SCLC and identified two potential biomarkers, high‐mobility group box 3 (HMGB3) and caspase‐10 (CASP10). Zinc finger homeobox 3 (ZFHX3) mutations were related to increased immune cell infiltration. Additionally, four distinct subtypes (nmf1‐4) with unique susceptibilities to available treatment options were identified, which were also confirmed in both cell lines and patient‐derived xenografts.

Lung cancer is the most common malignant tumor worldwide. In China, the incidence rate and mortality rate of lung cancer rank first among all cancers and are continuously on the rise.^2^ SCLC is the worst prognosis subtype of lung cancer with a 5‐year survival rate of only 5%. The main biological characteristics of SCLC are rapid growth, high metastasis rate, and high drug resistance as well as extensive‐stage SCLC accounts for about two‐thirds of all SCLC cases. The available treatment options for SCLC are significantly limited.^3^ The lack of comprehensive molecular characterization has impeded both basic and clinical advancements in SCLC.

Recently, the proteome‐centered multi‐omics strategy for clinical large cohort samples has comprehensively revealed the molecular features and potential therapeutic strategies of various tumors.^4^ Therefore, to comprehensively characterize SCLC, a systematic analysis of the proteogenomic features will be crucial for the precision medicine of SCLC.

In this study, authors first performed a multi‐omics analysis of the whole exome, transcriptome, proteome, and phosphorylated proteome. Somatic mutations and copy number deletions in TP53 and RB1 were the major genetic variants in SCLC. However, it is worth mentioning that the occurrence of mutations in TP53 and RB1, which are crucial oncogenes for the initiation of SCLC, was comparatively lower than what was observed in George's study (TP53:100% versus 72%; RB1: 90% versus 56%). This discrepancy may be attributed to variances in the composition of the study population (Chinese vs. Europeans) or variations in smoking habits (with 10% of subjects in Liu's study being non‐smokers vs. only 2% in George's study).^5^ Meanwhile, the frequency of mutations in ZFHX3 was higher in the Chinese population compared to Western populations.

Then, the authors performed the proteomic analysis by isobaric tandem mass tag labeling‐based global proteomics and phosphoproteomics and revealed various proteins, phosphorylation sites, and kinases (CHEK1, ATR, ATM, CDK2, and GSK3A) that displayed noteworthy alterations in the progression of SCLC. Furthermore, the HMGB3 (an unfavorable factor) and CASP10 (a favorable factor) were identified, which exhibited a significant association with the survival of patients with SCLC. Immunohistochemical analysis of an independent cohort confirmed their potential prognostic value in SCLC. Further biological experiments showed that HMGB3 could promote cell migration in SCLC through transcriptional regulation of cell‐junction‐related genes.

Subsequently, the researchers systematically analyzed the immune microenvironment characteristics of SCLC and performed immunosubtyping based on immune cell infiltration, and found that most of the SCLC belonged to the “cold‐tumor” enriched subtype with a poor prognosis, while only a few of them belonged to the “hot‐tumor”. Immune cold‐tumors exhibited higher neuroendocrine scores and lower immune scores than “hot‐tumor”. Meanwhile, immunity‐based signatures (like T cell markers, major histocompatibility complex molecules, and immune checkpoint molecules expression) were downregulated in immune‐cold than immune‐hot tumors, indicating that immunotherapy may benefit the immune‐hot subtype. Through integrated multi‐omics analysis, ZFHX3 mutations were significantly enriched in the “hot‐tumor” subtype. Patients with ZFHX3 mutations had a better response to combination chemotherapy with programmed cell death protein 1 or programmed death ligand 1 inhibitors.

Finally, the researchers used multi‐omics analysis to classify SCLC into four subtypes (nmf1‐4). Among these subtypes, nmf1 is the classical subtype, which exhibits rapid proliferation, suggesting the potential of a first‐line chemotherapy regimen based on cisplatin and etoposide. The nmf2 subtype exhibits significantly elevated DLL3 expression, suggesting the targeted therapy of DLL3 may be effective; and the nmf3 subtype exhibits significant activation of receptor tyrosine kinases (RTKs) pathway, suggesting that it may benefit from targeting RTKs. Patients with the nmf4 subtype exhibited specifically elevated MYC expression and MYC pathway activation, suggesting that they may benefit from aurora kinase inhibitors. Researchers also validated these targeted therapeutic strategies in a large‐scale patient‐derived xenograft and cell‐derived xenograft based on mouse models. This new molecular typing will also greatly deepen clinicians' understanding of the complexity and heterogeneity of solid tumors in order to formulate a more precise and effective therapeutic strategy. The new molecular typing mode was closely integrated with the clinicopathological data, which can reflect the changes at the molecular level, but also have a guiding significance for the personalized treatment.

Liu and colleagues conducted the first comprehensive proteomic and phosphorylated proteomic profiling of SCLC in Chinese populations, providing a fundamental framework for pathological mechanisms, prognostic assessment, molecular subtyping, and individualized therapeutic interventions. Moreover, the extensive dataset of this study will be a valuable resource for researchers in the field of SCLC.

However, the current cohort only includes resectable SCLC, which limits the application of metastatic SCLC, which accounts for 60%–65% of all patients with SCLC. Meanwhile, analysis of treatment‐naive patients lacks therapeutic drug data, limiting research to therapy‐related features. Bulk tumors and normal adjacent tissues are difficult to obtain for first‐diagnosed advanced SCLC patients. Therefore, multi‐omics analysis of liquid biopsy samples is promising for this rare and aggressive cancer. Integrating proteomics with other omics and technologies, such as single cell, spatial omics, metabolomics, or microbiomics from tissue biopsy and liquid biopsy of SCLC, could transform the unmet clinical need of SCLC (Figure 1).

**FIGURE 1:**
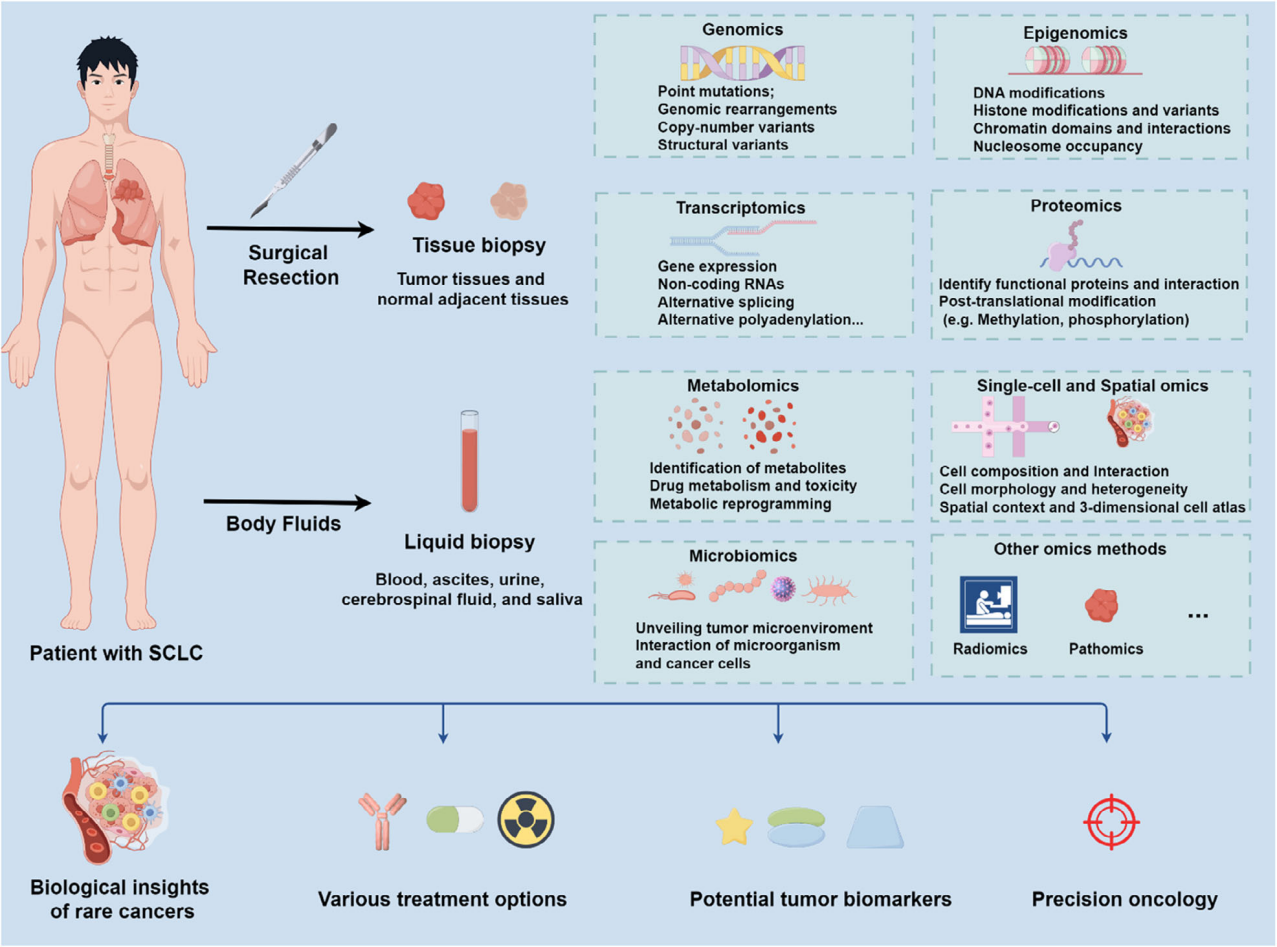
Integration of multi‐omics analysis into small‐cell lung cancer (SCLC) research. (created in www.figdraw.com).

Overall, this work is the first large‐scale proteomic and phosphorylated proteomic characterization of a clinical cohort of SCLC. These findings provide a theoretical basis for the analysis of pathological mechanisms, prognostic testing, molecular typing, and personalized treatment of SCLC, and the high‐quality data resources will provide support for the majority of basic and clinical researchers of SCLC and push forward the development of the field of SCLC research.

The advent of high‐throughput technologies has made multi‐omics a robust instrument in cancer research, enabling the concurrent investigation of cancer's molecular mechanisms across multiple levels. The recent advancements in multi‐omics have facilitated the shift from the conventional paradigm of life sciences. Specifically, there has been a transition from understanding diseases solely at the single‐omics level to the integration and analysis of multi‐omics data from large‐scale cohorts. Multi‐omics integrates diverse omics technologies, including genomics, transcriptomics, and proteomics, to provide comprehensive insights into the various malignant behaviors of cancer. As multi‐omics technology continues to evolve and mature, researchers are now able to address critical technical and scientific challenges in biomedicine, synthetic biology, and other disciplines through a systems biology approach. The significance of multi‐omics in cancer research extends beyond the aforementioned aspects, encompassing the elucidation of tumor‐microenvironment interactions, the unraveling of tumor metastasis and recurrence mechanisms, and the generation of novel insights for cancer prevention and treatment.

## AUTHOR CONTRIBUTIONS

Guo Zhao and Yuning Wang wrote the paper. Shuhang Wang and Ning Li revised the paper. All authors have read and approved the article.

## CONFLICT OF INTEREST STATEMENT

The authors declare no conflict of interest.

## ETHICS STATEMENT

Not applicable

## Data Availability

Not applicable.
